# A Game for Linear-time–Branching-time Spectroscopy

**DOI:** 10.1007/978-3-030-72016-2_1

**Published:** 2021-03-01

**Authors:** Benjamin Bisping, Uwe Nestmann

**Affiliations:** 8 Eindhoven University of Technology, Eindhoven, The Netherlands; 9 Aalborg University, Aalborg East, Denmark; Technische Universität Berlin, Berlin, Germany

**Keywords:** Process equivalence spectrum, Distinguishing formulas, Bisimulation games

## Abstract

We introduce a generalization of the bisimulation game that can be employed to find all relevant distinguishing Hennessy–Milner logic formulas for two compared finite-state processes. By measuring the use of expressive powers, we adapt the formula generation to just yield formulas belonging to the coarsest distinguishing behavioral preorders/equivalences from the linear-time–branching-time spectrum. The induced algorithm can determine the best fit of (in)equivalences for a pair of processes.

## 1 Introduction

Have you ever looked at two system models and wondered what would be the finest notions of behavioral equivalence to equate them—or, conversely: the coarsest ones to distinguish them? We run into this situation often when analyzing models and, especially, when devising examples for teaching. We then find ourselves fiddling around with whiteboards and various tools, each implementing different equivalence checkers. Would it not be nice to *decide all equivalences at once*?

*Example 1.* Consider the following CCS process $\mathsf{P}_1 = a.(b + c) + a.d$. It describes a machine that can be activated ($a$) and then either is in a state where one can choose from $b$ and c or where it can only be deactivated again ($d$). $\mathsf{P}_1$ shares a lot of properties with $\mathsf{P}_2 = a.(b + d) + a.(c + d)$. For example, they have the same traces (and the same completed traces). Thus, they are (completed) trace equivalent.

But they also have differences. For instance, $\mathsf{P}_1$ has a run where it executes $a$ and then cannot do $d$, while $\mathsf{P}_2$ does not have such a run. Hence, they are *not failure equivalent*. Moreover, $\mathsf{P}_1$ may perform $a$ and then choose from $b$ and $c$, and $\mathsf{P}_2$ cannot. This renders the two processes also *not simulation equivalent*. Failure equivalence and simulation equivalence are incomparable—that is, neither one follows from the other one. *Both* are coarsest ways of telling the processes apart. Other inequivalences, like bisimulation inequivalence, are implied by both.

In the following, we present a uniform game-based way of finding the most fitting notions of (in)equivalence for process pairs like in Ex. 1.

Our approach is based on the fact that notions of process equivalence can be characterized by two-player games. The defender's winning region in the game corresponds to pairs of equivalent states, and the attacker's winning strategies correspond to distinguishing formulas of Hennessy–Milner logic (HML).

Each notion of equivalence in van Glabbeek's famous linear-time–branching-time spectrum [10] can be characterized by a subset of HML with specific distinguishing power. Some of the notions are incomparable. So, often a process pair that is equivalent with respect to one equivalence, is distinguished by a set of slightly coarser or incomparable equivalences, without any one of them alone being *the* coarsest way to distinguish the pair. As with the spectrum of light where a mix of wave lengths shows to us as a color, there is a "mix" of distinguishing capabilities involved in establishing whether a specific equivalence is finest. Our algorithm is meant to analyze what is in the mix.

**Contributions.** This paper makes the following contributions:

- We introduce a *special bisimulation game* that neatly characterizes the distinguishing formulas of HML for pairs of states in finite transition systems (Subsection 3.1 and 3.2).
- We show how to *enumerate the relevant distinguishing formulas* using the attacker's winning region (Subsection 3.3).
- We give a way of constructing a *finite set of distinguishing formulas guaranteed to contain observations of the weakest possible observation languages*, which can be seen as a "spectroscopy" of the differences between two processes (Subsection 3.4).
- We present a small *web tool that is able to run the algorithm on finite-state processes* and output a visual representation of the game (Section 4). We also report on the distinctions it finds for all the finitary examples from the report version of the linear-time–branching-time spectrum [12].

We frame the contributions by a roundtrip through the basics of HML, games and the spectrum (Section 2), a discussion of related work (Section 5), and concluding remarks on future lines of research (Section 6).

## 2 Preliminaries: HML, Games, and the Spectrum

We use the concepts of transition systems, games, observations, and notions of equivalence, largely due to the wake of Hennessy and Milner's seminal paper [14].

### 2.1 Transition Systems and Hennessy–Milner Logic

*Labeled transition systems* capture a discrete world view, where there is a current state and a branching structure of possible state changes to future states.

**Definition 1 (Labeled transition system).** *A* labeled transition system *is a tuple* $\mathcal{S} = (\mathcal{P}, \Sigma, \rightarrow)$ *where* $\mathcal{P}$ *is the set of* states*,* $\Sigma$ *is the set of* actions*, and* $\rightarrow \subseteq \mathcal{P} \times \Sigma \times \mathcal{P}$ *is the* transition relation*.*

*Hennessy–Milner logic* [14] describes finite *observations* (or "tests") that one can perform on such a system.

**Definition 2 (Hennessy–Milner logic).** *Given an alphabet $\Sigma$, the syntax of* Hennessy–Milner logic *formulas,* $\mathsf{HML}[\Sigma]$, *is inductively defined as follows:*

**Observations** *If* $\varphi \in \mathsf{HML}[\Sigma]$ *and* $a \in \Sigma$, *then* $\langle a \rangle \varphi \in \mathsf{HML}[\Sigma]$.
**Conjunctions** *If* $\varphi_i \in \mathsf{HML}[\Sigma]$ *for all* $i$ *from an index set* $I$, *then* $\bigwedge_{i \in I} \varphi_i \in \mathsf{HML}[\Sigma]$.
**Negations** *If* $\varphi \in \mathsf{HML}[\Sigma]$, *then* $\neg\varphi \in \mathsf{HML}[\Sigma]$.

We often just write $\bigwedge\{\varphi_0, \varphi_1, ...\}$ for $\bigwedge_{i \in I} \varphi_i$. $\mathsf{T}$ denotes $\bigwedge\emptyset$, the nil-element of the syntax tree, and $\langle a \rangle$ is a short-hand for $\langle a \rangle\mathsf{T}$. Let us also implicitly assume that formulas are flattened in the sense that conjunctions do not contain other conjunctions as immediate subformulas. We will sometimes talk about the syntax tree height of a formula and consider the height of $\mathsf{T}$ to equal 0.

Intuitively, $\langle a \rangle \varphi$ means that one can observe a system transition labeled by $a$ and then continue to make observation(s) $\varphi$. Conjunction and negation work as known from propositional logic. We will provide a common game semantics for $\mathsf{HML}$ in the following subsection.

### 2.2 Games Semantics of HML

Let us fix some notions for *Gale-Stewart-style reachability games* where the defender wins all infinite plays.

**Definition 3 (Games).** *A* simple reachability game $\mathcal{G}[g_0] = (G, G_d, \rightarrowtail, g_0)$ *consists of*

- *a set of* game positions $G$, *partitioned into*
  - *a set of* defender positions $G_d \subseteq G$
  - *and* attacker positions $G_a := G \setminus G_d$,
- *a graph of* game moves $\rightarrowtail \subseteq G \times G$, *and*
- *an* initial position $g_0 \in G$.

**Definition 4 (Plays and wins).** *We call the paths* $g_0 g_1 ... \in G^\infty$ *with* $g_i \rightarrowtail g_{i+1}$ plays *of* $\mathcal{G}[g_0]$. *The defender* wins *infinite plays. If a finite play* $g_0 \dots g_n \not\rightarrowtail$ *is stuck, the stuck player loses: The defender wins if* $g_n \in G_a$, *and the attacker wins if* $g_n \in G_d$.

**Definition 5 (Strategies and winning strategies).** *A (positional, nondeterministic)* strategy *is a subset of the moves,* $F \subseteq \rightarrowtail$. *If (fairly) picking elements of strategy* $F$ *ensures a player to win,* $F$ *is called a* winning strategy *for this player. The player with a winning strategy for* $\mathcal{G}[g_0]$ *is said to* win $\mathcal{G}[g_0]$.

**Definition 6 (Winning regions).** *The set* $W_a \subseteq G$ *of all positions* $g$ *where the attacker wins* $\mathcal{G}[g]$ *is called the* attacker winning region *(defender winning region* $W_d$ *analogous).*

All Gale-Stewart-style reachability games are *determined*, that is, $W_a \cup W_d = G$. The winning regions of finite simple reachability games can be computed in linear time of the number of game moves (cf. [13]). This is why the spectroscopy game of this paper can easily be used in algorithms. It derives from the following game.

**Definition 7 (HML game).** *For a transition system* $\mathcal{S} = (\mathcal{P}, \Sigma, \rightarrow)$, *the* HML game $\mathcal{G}^{\mathcal{S}}_{\mathsf{HML}}[g_0] = (G, G_d, \rightarrowtail, g_0)$ *is played on* $G = \mathcal{P} \times \mathsf{HML}[\Sigma]$, *where the defender controls observations and negated conjunctions, that is* $(p, \langle a \rangle \varphi) \in G_d$ *and* $(p, \neg \bigwedge_{i \in I} \varphi_i) \in G_d$ *(for all* $\varphi, p, I$*), and the attacker controls the rest. There are five kinds of moves:*

- $(p, \langle a \rangle \varphi) \quad \rightarrowtail \quad (p', \varphi) \quad$ *if* $p \xrightarrow{a} p'$,
- $(p, \neg \langle a \rangle \varphi) \quad \rightarrowtail \quad (p', \neg \varphi) \quad$ *if* $p \xrightarrow{a} p'$,
- $(p, \bigwedge_{i \in I} \varphi_i) \quad \rightarrowtail \quad (p, \varphi_i) \quad$ *with* $i \in I$,
- $(p, \neg \bigwedge_{i \in I} \varphi_i) \quad \rightarrowtail \quad (p, \neg \varphi_i) \quad$ *with* $i \in I$, *and*
- $(p, \neg\neg\varphi) \quad \rightarrowtail \quad (p, \varphi)$.

Like in other logical games in the Ehrenfeucht–Fraïssé tradition, the attacker plays the conjunctions and universal quantifiers, whereas the defender plays the disjunctions and existential quantifiers. For instance, $(p, \langle a \rangle \varphi)$ is declared as defender position, since $\langle a \rangle \varphi$ is meant to become true precisely if *there exists* a state $p'$ reachable $p \xrightarrow{a} p'$ where $\varphi$ is true.

As every move strictly reduces the height of the formula, the game must be finite-depth (and cycle-free), and, for image-finite systems and formulas, also finite. It is determined and the following semantics is total.

**Definition 8 (HML semantics).** *For a transition system* $\mathcal{S} = (\mathcal{P}, \Sigma, \rightarrow)$, *the* semantics of HML *is given by defining that* $\varphi$ *is true at* $p$ *in* $\mathcal{S}$, *written* $[\![\varphi]\!]^{\mathcal{S}}_p$, *iff the defender wins* $\mathcal{G}^{\mathcal{S}}_{\mathsf{HML}}[(p, \varphi)]$.

*Example 2.* Continuing Ex. 1, $[\![\langle a \rangle \neg \langle d \rangle \mathsf{T}]\!]^{\mathsf{CCS}}_{\mathsf{P}_2}$ is false: No matter whether the defender plays to $(b + d, \neg \langle d \rangle \mathsf{T})$ or to $(c + d, \neg \langle d \rangle \mathsf{T})$, the attacker wins by moving to the stuck defender position $(\mathbf{0}, \neg \mathsf{T})$. (Recall that $\mathsf{T}$ is the empty conjunction and that $\mathbf{0}$ is the completed process!)

### 2.3 The Spectrum of Behavioral Equivalences

**Definition 9 (Distinguishing formula).** *A formula* $\varphi$ distinguishes *state* $p$ *from* $q$ *iff* $[\![\varphi]\!]_p$ *is true and* $[\![\varphi]\!]_q$ *is not.*[1]

*Example 3.* $\langle a \rangle \neg \langle d \rangle \mathsf{T}$ distinguishes $\mathsf{P}_1$ from $\mathsf{P}_2$ in Ex. 1 (but not the other way around). $\langle a \rangle \bigwedge \{\langle b \rangle \mathsf{T}, \langle d \rangle \mathsf{T}\}$ distinguishes $\mathsf{P}_2$ from $\mathsf{P}_1$.

**Definition 10 (Observational preorders and equivalences).** *A set of observations,* $\mathcal{O}_X \subseteq \mathsf{HML}[\Sigma]$, preorders *two states* $p, q$, *written* $p \sqsubseteq_X q$, *iff no formula* $\varphi \in \mathcal{O}_X$ *distinguishes* $p$ *from* $q$. *If* $p \sqsubseteq_X q$ *and* $q \sqsubseteq_X p$, *then the two are* $X$*-equivalent, written* $p \equiv_X q$.

[1] In the following, we usually leave the transition system $\mathcal{S}$ implicit.

**Definition 11 (Linear-time–branching-time languages [12]).** *The linear-time–branching-time spectrum is a lattice of observation languages (and of entailed process preorders and equivalences). Every observation language $\mathcal{O}_X$ can perform trace observations, that is,* $\mathsf{T} \in \mathcal{O}_X$ *and, if* $\varphi \in \mathcal{O}_X$*, then* $\langle a \rangle \varphi \in \mathcal{O}_X$*. At the more linear-time side of the spectrum we have:*

- trace observations $\mathcal{O}_T$: *Just trace observations,*
- failure observations $\mathcal{O}_F$: $\bigwedge_{i \in I} \neg \langle a_i \rangle \in \mathcal{O}_F$,
- readiness observations $\mathcal{O}_R$: $\bigwedge_{i \in I} \varphi_i \in \mathcal{O}_R$ *with each* $\varphi_i$ *of form* $\neg \langle a_i \rangle$ *or* $\langle a_i \rangle$,
- failure trace observations $\mathcal{O}_{FT}$: $\bigwedge_{i \in I} \varphi_i \in \mathcal{O}_{FT}$ *with* $\varphi_0 \in \mathcal{O}_{FT}$ *and, for* $i > 0$, $\varphi_i = \neg \langle a_i \rangle$,
- ready trace observations $\mathcal{O}_{RT}$: $\bigwedge_{i \in I} \varphi_i \in \mathcal{O}_{RT}$ *with* $\varphi_0 \in \mathcal{O}_{RT}$ *and, for* $i > 0$, $\varphi_i$ *of form* $\neg \langle a_i \rangle$ *or* $\langle a_i \rangle$,
- impossible futures $\mathcal{O}_{IF}$: $\bigwedge_{i \in I} \neg \varphi_i \in \mathcal{O}_{IF}$ *with all* $\varphi_i \in \mathcal{O}_T$*, and*
- possible futures $\mathcal{O}_{PF}$: $\bigwedge_{i \in I} \varphi_i \in \mathcal{O}_{PF}$ *with all* $\varphi_i \in \{\neg \psi_i, \psi_i\}$ *and* $\psi_i \in \mathcal{O}_T$.[2]

*At the more branching-time side, we have simulation observations. Every simulation observation language $\mathcal{O}_{XS}$, has full conjunctive capacity, that is, if* $\varphi_i \in \mathcal{O}_{XS}$ *for all* $i \in I$*, then* $\bigwedge_{i \in I} \varphi_i \in \mathcal{O}_{XS}$*.*

- simulation observations $\mathcal{O}_{1S}$: *Just simulation (and trace) observations,*
- $n$-nested simulation observations $\mathcal{O}_{nS}$: $\neg \varphi \in \mathcal{O}_{nS}$ *with* $\varphi \in \mathcal{O}_{(n-1)S}$,
- ready simulation observations $\mathcal{O}_{RS}$: $\neg \langle a \rangle \in \mathcal{O}_{RS}$*, and*
- bisimulation observations $\mathcal{O}_B$: *The same as* $\mathcal{O}_{\infty S}$*, which is exactly* $\mathsf{HML}[\Sigma]$.

The observation languages of the spectrum differ in how many of the syntactic features of HML one will encounter when descending into a formula's syntax tree. We will come back to this in Subsection 3.4.

Note that we consider $\bigwedge\{\varphi\}$ to be an alias for $\varphi$. With this aliasing, all the listed observation languages are *closed* in the sense that all subformulas of an observation are themselves part of that language. They thus are *inductive* in the sense that all observations must be built from observations of the same language with lower syntax tree height.

## 3 Distinguishing Formula Games

This section introduces our main contribution: the spectroscopy game (Def. 13), and how to build all interesting distinguishing HML formulas from its winning region (Def. 14). To justify our construction and to prove that we indeed find distinguishing formulas (Thm. 1), let us first examine the formula preorder game (Def. 12), which is closer to the problem whether formulas are (non-)distinguishing.

[2] Like Kučera and Esparza [17], who studied the properties of "good" observation languages, we glimpse over completed trace, completed simulation and possible worlds observations here, because these observations need a special exhaustive $\bigwedge_{a \in \Sigma} \varphi$. While it could be provided for with additional operators, it would add another case in each of the upcoming definitions and would break the closure property of observation languages, without giving much in return.

### 3.1 The Formula Preorder Game

Def. 10 entails a straightforward way of turning the problem whether a set of observations $\mathcal{O} \subseteq \mathcal{O}_X$ preorders two states $p, q$ into a game: Have the attacker pick a supposedly distinguishing formula $\varphi \in \mathcal{O}$, and then have the defender choose whether to play the HML game (Def. 7) for $[\![\neg\varphi]\!]_p$ or for $[\![\varphi]\!]_q$. This direct route will yield infinite games for infinite $\mathcal{O}$—and all the languages from Def. 11 are infinite!

To bypass the infinity issue, we will introduce a variation of this game *where the attacker gradually chooses their attacking formula.* In particular, this means that the attacker now decides which observations to play. In return, the defender does not need to pick a side in the beginning and may postpone the decision where (on the right-hand side) an observation leads. Postponing decisions here means that the defender may play non-deterministically, moving to multiple states at once. The mechanics are analogous to the standard powerset construction when transforming non-deterministic finite automata into deterministic ones.

**Definition 12 (Formula preorder game).** *For a transition system* $\mathcal{S} = (\mathcal{P}, \Sigma, \rightarrow)$ *and a set of observations* $\mathcal{O}_X$*, the* formula preorder game $\mathcal{G}^{\mathcal{S}}_X[g_0] = (G, G_d, \rightarrowtail, g_0)$ *consists of*

- attacker positions $(p, Q, \mathcal{O})_{\mathtt{a}} \in G_a$ *with* $p \in \mathcal{P}$*,* $Q \in 2^{\mathcal{P}}$*, and* $\mathcal{O} \subseteq \mathcal{O}_X$*,*
- defender conjunction positions $(p, Q, \mathcal{O})^{\wedge}_{\mathtt{d}} \in G_d$ *where the defender has to answer to conjunction challenges, and*
- defender negation positions $(p, Q, \mathcal{O})^{\neg}_{\mathtt{d}} \in G_d$ *where the defender has to answer to negation challenges,*

*and five kinds of moves*

- observation moves $(p, Q, \mathcal{O})_{\mathtt{a}} \rightarrowtail (p', Q', \mathcal{O}')_{\mathtt{a}}$
  *if* $p \xrightarrow{a} p'$ *with* $Q' = \{q' \mid \exists q \in Q.\, q \xrightarrow{a} q'\}$ *and* $\mathcal{O}' = \{\varphi \mid \langle a \rangle \varphi \in \mathcal{O}\}$*,*
- conjunct challenges $(p, Q, \mathcal{O})_{\mathtt{a}} \rightarrowtail (p, Q, \{\varphi_i \mid i \in I\})^{\wedge}_{\mathtt{d}}$
  *if* $\bigwedge_{i \in I} \varphi_i \in \mathcal{O}$*,*
- conjunct answers $(p, Q, \mathcal{O})^{\wedge}_{\mathtt{d}} \rightarrowtail (p, \{q\}, \mathcal{O})_{\mathtt{a}}$
  *if* $q \in Q$*,*
- negation challenges $(p, Q, \mathcal{O})_{\mathtt{a}} \rightarrowtail (p, Q, \{\varphi\})^{\neg}_{\mathtt{d}}$
  *if* $\neg\varphi \in \mathcal{O}$*, and*
- negation answers $(p, Q, \mathcal{O})^{\neg}_{\mathtt{d}} \rightarrowtail (q, \{p\}, \mathcal{O})_{\mathtt{a}}$
  *if* $q \in Q$*.*

The formula preorder game precisely characterizes whether an observation language is distinguishing:

**Lemma 1.** *For a closed observation language* $\mathcal{O}_X$*, the formula preorder game* $\mathcal{G}^{\mathcal{S}}_X[(p, Q, \mathcal{O})_{\mathtt{a}}]$ *with* $\mathcal{O} \subseteq \mathcal{O}_X$ *is won by the defender precisely if, for every observation* $\varphi \in \mathcal{O}$ *with* $[\![\varphi]\!]_p$*, there is a* $q \in Q$ *such that* $[\![\varphi]\!]_q$*.*

*Proof (Sketch).* By induction over the height of formulas in $\mathcal{O}_X$ with arbitrary $p$ and $Q$, and strengthening the induction predicate to not only consider $\varphi$ but also partial conjunctions $\bigwedge \mathcal{O}''$ with $\mathcal{O}'' \subseteq \mathcal{O}'$ whenever $\varphi = \bigwedge \mathcal{O}'$. To prove the right-to-left direction, exploiting the determinacy of the game is convenient.

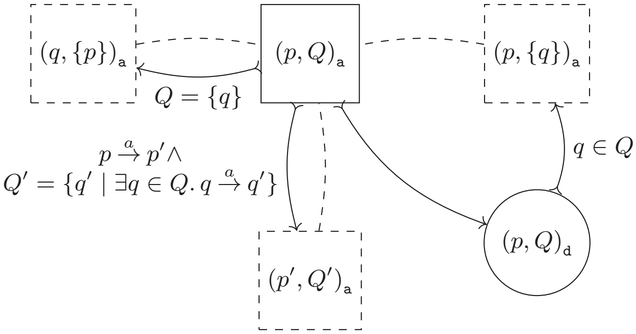

**Figure 1.** Schematic spectroscopy game $\mathcal{G}_\triangle$ of Def. 13. Boxes stand for attacker positions, circles for defender positions, arrows for moves. From the dashed boxes, the moves are analogous to the ones of the connected solid positions.

### 3.2 The Spectroscopy Game

Let us now remove the formulas from the formula game (Def. 12). The idea is to look at the game for the whole of HML, called $\mathcal{G}_B$. Only attack moves in the formula game change the current set of observations, and they are completely guided by the context-free grammar of HML (Def. 2). Therefore, we can[3] assume $\mathcal{O}$ to equal $\mathsf{HML}[\Sigma]$ in every reachable position of $\mathcal{G}_B$. Effectively, $\mathcal{O}$ can be canceled out of the game, without losing any information. We call the remaining game the "spectroscopy game." Figure 1 gives a graphical representation.

**Definition 13 (Spectroscopy game).** *For a transition system $\mathcal{S} = (\mathcal{P}, \Sigma, \rightarrow)$, the L-labeled* spectroscopy game $\mathcal{G}_\triangle^{\mathcal{S}}[g_0] = (G, G_d, \dot{\rightarrowtail}, g_0)$ *with $L = \{\neg, \wedge, *, \langle a\rangle\}$ consists of*

- attacker positions $(p,Q)_{\mathtt{a}} \in G_a$ *with $p \in \mathcal{P}$, $Q \in 2^{\mathcal{P}}$,*
- defender positions $(p,Q)_{\mathtt{d}} \in G_d$ *where the defender has to answer to conjunction challenges,*

*and four kinds of moves:*

- observation moves $\quad (p,Q)_{\mathtt{a}} \overset{\langle a\rangle}{\rightarrowtail} (p', \{q' \mid \exists q \in Q.\, q \xrightarrow{a} q'\})_{\mathtt{a}} \quad$ *if $p \xrightarrow{a} p'$*
- conjunct challenges $\quad (p,Q)_{\mathtt{a}} \overset{\wedge}{\rightarrowtail} (p,Q)_{\mathtt{d}}$,
- conjunct answers $\quad (p,Q)_{\mathtt{d}} \overset{*}{\rightarrowtail} (p,\{q\})_{\mathtt{a}} \quad$ *if $q \in Q$, and*
- negation moves $\quad (p,\{q\})_{\mathtt{a}} \overset{\neg}{\rightarrowtail} (q,\{p\})_{\mathtt{a}}$.

We have already introduced two tricks in this definition to ease formula reconstruction in the next subsection. (1) The attack moves are labeled with the

[3] To be precise: Finite conjunctions may only lead to *arbitrarily large* subsets of $\mathsf{HML}[\Sigma]$. If the attacker has a way of winning by playing a conjunction, we can as well approximate this move as playing $\bigwedge\mathsf{HML}$.

syntactic constructs from which they originate. This does not change expressive power. (2) Negation moves are restricted to situations where $Q = \{q\}$. After all, winning attacker strategies will pay attention to only playing a negation after minimizing the odds of being put on a bad position, anyways.

Note that, like in the formula game with arbitrary-depth formulas, the attacker could force infinite plays by cycling through conjunction moves (and also negation moves). However, they will not do this, as infinite plays are won by the defender.

**Lemma 2.** *The spectroscopy game $\mathcal{G}_\triangle[(p, \{q\})_\mathsf{a}]$ is won by the defender precisely if $p$ and $q$ are bisimilar.*

This fact is a corollary of the well-known Hennessy–Milner theorem ($\mathsf{HML}$ characterizes bisimilarity), given that $\mathcal{G}_\triangle$ is constructed as a simplification of $\mathcal{G}_B$.

Comparing $\mathcal{G}_\triangle$ to the standard bisimulation game from the literature (with symmetry moves, see e.g. [3]), we can easily transfer attacker strategies from there. In the standard game, the attacker will play $(p, q) \rightarrowtail (a, p', q)$ with $p \xrightarrow{a} p'$ and the defender has to answer by $(a, p', q) \rightarrowtail (p', q')$ with $q \xrightarrow{a} q'$. In the spectroscopy game, the attacker can enforce analogous moves by playing $(p, \{q\})_\mathsf{a} \overset{\langle a \rangle}{\rightarrowtail} (p', Q')_\mathsf{a} \overset{\wedge}{\rightarrowtail} (p', Q')_\mathsf{d}$, which will make the defender pick $(p', Q')_\mathsf{d} \overset{*}{\rightarrowtail} (p', \{q'\})_\mathsf{a}$.

The opposite direction of transfer is not so easy, as the attacker has more ways of winning in $\mathcal{G}_\triangle$. But this asymmetry is precisely why we have to use the spectroscopy game instead of the standard bisimulation game if we want to learn about, for example, interesting failure-trace attacks.

Due to the subset construction over $\mathcal{P}$, the game size clearly is exponential in the size of the state space. Going exponential is necessary, as we want to also characterize weaker preorders like the trace preorder, where exponential $\mathcal{P}$-subset or $\Sigma^*$-word constructions cannot be circumvented. However, for moderate real-world systems, such constructions will not necessarily show their full exponential blow-up (cf. [6]).

For concrete implementations, the subset construction also means that the costs of storing game nodes and of comparing two nodes is linear in the state space size. Complexity-wise this factor is dominated by the overall exponentialities.

### 3.3 Building Distinguishing Formulas from Attacker Strategies

**Definition 14 (Strategy formulas).** *Given an attacker strategy $F \subseteq (G_a \times L \times G)$ for the spectroscopy game $\mathcal{G}_\triangle$, the set of* strategy formulas*, $\mathsf{Strat}_F(g_a)$, is inductively defined by:*

- *If $\varphi \in \mathsf{Strat}_F(g'_a)$ and $(g_a, \langle b \rangle, g'_a) \in F$, then $\langle b \rangle \varphi \in \mathsf{Strat}_F(g_a)$,*
- *if $\varphi \in \mathsf{Strat}_F(g'_a)$ and $(g_a, \neg, g'_a) \in F$, then $\neg\varphi \in \mathsf{Strat}_F(g_a)$, and*
- *if $\varphi_{g'_a} \in \mathsf{Strat}_F(g'_a)$ for all $g'_a \in I = \{g'_a \mid g_d \overset{*}{\rightarrowtail}_\triangle g'_a\}$, and $(g_a, \wedge, g_d) \in F$, then $\bigwedge_{g'_a \in I} \varphi_{g'_a} \in \mathsf{Strat}_F(g_a)$.*

*Example 4.* The attacks $(\mathsf{P}_1, \{\mathsf{P}_2\})_\mathsf{a} \overset{\langle a \rangle}{\rightarrowtail} (b + c, \{b + d, c + d\})_\mathsf{a} \overset{\wedge}{\rightarrowtail} \overset{*}{\rightarrowtail} \overset{\neg}{\rightarrowtail} \overset{\langle d \rangle}{\rightarrowtail} (\mathbf{0}, \emptyset)_\mathsf{a} \overset{\wedge}{\rightarrowtail}$ give rise to the formula $\langle a \rangle \bigwedge \{\neg \langle d \rangle \mathsf{T}\}$, which can be written as $\langle a \rangle \neg \langle d \rangle$.

**Definition 15 (Winning strategy graph).** *Given the attacker winning region* $W_a$ *and a starting position* $g_0 \in W_a$*, the* attacker winning strategy graph $F_a$ *is the subset of the* $\rightarrowtail$*-graph that can be visited from* $g_0$ *when following all* $\rightarrowtail$*-edges unless they lead out of* $W_a$*.*

This graph can be cyclic. However, if the attacker plays inside their winning region according to $F_a$, they will always have paths to their final winning positions. So even though the attacker could loop (and thus lose), they can always end the game and *win* in the sense of Def. 5.

**Theorem 1.** *If* $W_a$ *is the attacker winning region of the spectroscopy game* $\mathcal{G}_\triangle$*, every* $\varphi \in \mathsf{Strat}_{F_a}((p, \{q\})_\mathtt{a})$ *distinguishes* $p$ *from* $q$*.*

*Proof.* Due to Lem. 1, it suffices to show that $\varphi \in \mathsf{Strat}_{F_a}((p, Q)_\mathtt{a})$ implies that the attacker wins $\mathcal{G}_B[(p, Q, \{\varphi\})]$. We proceed by induction on the structure of $\mathsf{Strat}_{F_a}$ with arbitrary $p, Q$.

- Assume $\varphi \in \mathsf{Strat}_{F_a}((p', Q')_\mathtt{a})$ and $((p, Q)_\mathtt{a}, \langle b\rangle, (p', Q')_\mathtt{a}) \in F_a$. By induction hypothesis, the attacker wins $\mathcal{G}_B[(p', Q', \{\varphi\})]$. By moving there, the attacker also wins $\mathcal{G}_B[(p, Q, \{\langle b\rangle\varphi)\}]$, which must be a valid move as $F_a$ is a strategy for $\mathcal{G}_\triangle$.
- Assume $\varphi \in \mathsf{Strat}_{F_a}((p', Q')_\mathtt{a})$ and $((p, Q)_\mathtt{a}, \neg, (p', Q')_\mathtt{a}) \in F_a$. By induction hypothesis, the attacker wins $\mathcal{G}_B[(p', Q', \{\varphi\})]$. By the construction of $\mathcal{G}_\triangle$, $Q = \{p'\}$. So the attacker can win $\mathcal{G}_B[(p, Q, \{\neg\varphi\})]$ by moving to this position (with the defender having no choice when picking from $Q$).
- Assume $\varphi_{g'_a} \in \mathsf{Strat}_{F_a}(g'_a)$ for all $g'_a = (p', \{q'\})_\mathtt{a} \in I = \{g'_a \mid g_d \overset{*}{\rightarrowtail}_\triangle g'_a\}$, and $((p, Q)_\mathtt{a}, \wedge, g_d) \in F_a$. Due to the construction of $\mathcal{G}_\triangle$, $Q = \{q' \mid (p', \{q'\})_\mathtt{a} \in I\}$ and $p' = p$. By induction hypothesis, the attacker wins all $\mathcal{G}_B[(p', \{q'\}, \{\varphi_{g'_a}\})]$ and, as they can always focus on consuming just one formula, also all $\mathcal{G}_B[(p, \{q'\}, \{\varphi_{g''_a} \mid g''_a \in I\})]$. This matches all the positions the defender can move to after $(p, Q, \{\varphi_{g''_a} \mid g''_a \in I\})_\mathtt{d}$. Moving there, the attacker wins $\mathcal{G}_B[(p, Q, \{\bigwedge_{g'_a \in I} \varphi_{g'_a}\})]$.

Note that the theorem is only one-way, as every distinguishing formula can neutrally be extended by saying that some additional clause that is true for *both* processes does hold. Def. 14 will not find such bloated formulas.

Due to cycles in the game graph, $\mathsf{Strat}_{F_a}$ will usually yield infinitely many formulas. But we can become finite by injecting some way of discarding long formulas that unfold negation cycles or recursions of the underlying transition system. The next section will discuss how to do this without discarding the formulas that are interesting from the point of view of the spectrum.

### 3.4 Retrieving Cheapest Distinguishing Formulas

In our quest for the coarsest behavioral preorders (or equivalences) distinguishing two states, we actually are only interested in the ones that are part of the *smallest*

*observation languages* from the spectrum (Def. 11). We can think of the amount of HML-expressiveness used by a formula as its *price*.

Let us look at the price structure of the spectrum from Def. 11. Table 1 gives an overview of how many syntactic HML-features the observation languages may use at most. (If formulas use fewer, they still are considered part of that observation language.) So, we are talking *budgets*, in the price analogy.

**Conjunctions:** How often may one run into a conjunction when descending down the syntax tree. Negations in the beginning or following an observation are counted as implicit conjunctions.

**Positive deep branches:** How many positive deep branches may appear in each conjunction? We call subformulas of the form $\langle a \rangle$ or $\neg\langle a \rangle$ *flat branches*, and the others *deep branches*.

**Positive flat branches:** How many positive flat branches may appear in each conjunction?[4]

**Negations:** How many negations may be visited when descending?

**Negations height:** How high can the syntax trees under each negation be?

We say that a formula $\varphi_1$ *dominates* $\varphi_2$ if $\varphi_1$ has lower or equal values than $\varphi_2$ in each dimension of the metrics with at least one entry strictly lower. Let us note the following facts:

[4] There is a special case for failure-traces where 1 positive flat branch may be counted as deep, if there are no other deep branches. Hence the * in Table 1.

**Table 1.** Dimensions of observation expressiveness.

| Observations | Conjunctions | Positive deep br. | Positive flat br. | Negations | Negation height |
|---|---|---|---|---|---|
| trace $\mathcal{O}_T$ | 0 | 0 | 0 | 0 | 0 |
| failure $\mathcal{O}_F$ | 1 | 0 | 0 | 1 | 1 |
| readiness $\mathcal{O}_R$ | 1 | 0 | $\infty$ | 1 | 1 |
| failure-trace $\mathcal{O}_{FT}$ | $\infty$ | 1 | 0* | 1 | 1 |
| ready-trace $\mathcal{O}_{RT}$ | $\infty$ | 1 | $\infty$ | 1 | 1 |
| impossible-future $\mathcal{O}_{IF}$ | 1 | 0 | 0 | 1 | $\infty$ |
| possible-future $\mathcal{O}_{PF}$ | 1 | $\infty$ | $\infty$ | 1 | $\infty$ |
| ready-simulation $\mathcal{O}_{RS}$ | $\infty$ | $\infty$ | $\infty$ | 1 | 1 |
| $(n+1)$-nested-simulation $\mathcal{O}_{(n+1)S}$ | $\infty$ | $\infty$ | $\infty$ | $n$ | $\infty$ |
| bisimulation $\mathcal{O}_B$ | $\infty$ | $\infty$ | $\infty$ | $\infty$ | $\infty$ |

```
def game_spectroscopy(𝒮, p₀, q₀):
    𝒢△^𝒮 = (G, G_a, ↣) := construct_spectroscopy_game(𝒮)
    W_a := compute_winning_region(𝒢△^𝒮)
    if (p₀, {q₀})_a ∈ W_a :
        F_a := winning_graph(𝒢△^𝒮, W_a, (p₀, {q₀})_a)
        strats[] := ∅
        todo := [(p₀, {q₀})_a]
        while todo ≠ []:
            g := todo.dequeue()
            sg := strats[g]
            if sg = undefined :
                strats[sg] := ∅
            gg′ := {g′ | (g, ·, g′) ∈ F_a ∧ strats(g′) = undefined}
            if gg′ = ∅ :
                sg′ = nonDominatedOrIF(Strat′_{F_a,strats}(g))
                if sg ≠ sg′ :
                    strats(g) := sg′
                    todo.enqueueEachEnd({g* | (g*, ·, g) ∈ F_a ∧ g* ∉ todo})
            else:
                todo.enqueueEachFront(gg′)
        return strats((p₀, {q₀})_a)
    else:
        ℛ := {(p, q) | (p, {q})_a ∈ G_a \ W_a}
        return ℛ
```

Algorithm 1: Spectroscopy procedure.

1. When formulas are constructed recursively, like the strategy formulas in Def. 14, they can only contribute to dominating (i.e. more expensive) or equivalently valued formulas with respect to the metrics.
2. Formulas can be incomparable. For example, $\langle a\rangle\bigwedge\{\langle b\rangle, \langle c\rangle\}$ and $\langle a\rangle\neg\langle a\rangle$, corresponding to coordinates (1,0,2,0,0) and (1,0,0,1,1), are incomparable.
3. A locally more expensive formula may pay off as part of a bigger global formula. For example, if two states are distinguished by $\neg\langle a\rangle$ and $\langle b\rangle$, the dominated formula $\neg\langle a\rangle$ may later be handy to construct a (comparably cheap) failure formula.

These observations justify our algorithm to prune all formulas from the set $\mathsf{Strat}_{F_a}(g)$ that are dominated with respect to the metrics by any other formula in this set, unless they are *impossible trace futures* of the form $\neg\langle a_1\rangle\langle a_2\rangle\ldots$. We moreover add formula height in terms of observations as a dimension in the metric, which leads to loop unfoldings being dominated by the shorter paths.

Algorithm 1 shows all the elements in concert. It constructs the spectroscopy game $\mathcal{G}_\triangle^\mathcal{S}$ (Def. 13) and computes its attacker winning strategy graph $F_a$ (Def. 15). If the attacker cannot win, the algorithm returns a bisimulation relation. Otherwise, it constructs the distinguishing formulas: It keeps a map strats of strategy formulas that have been found so far and a list of game positions todo that have

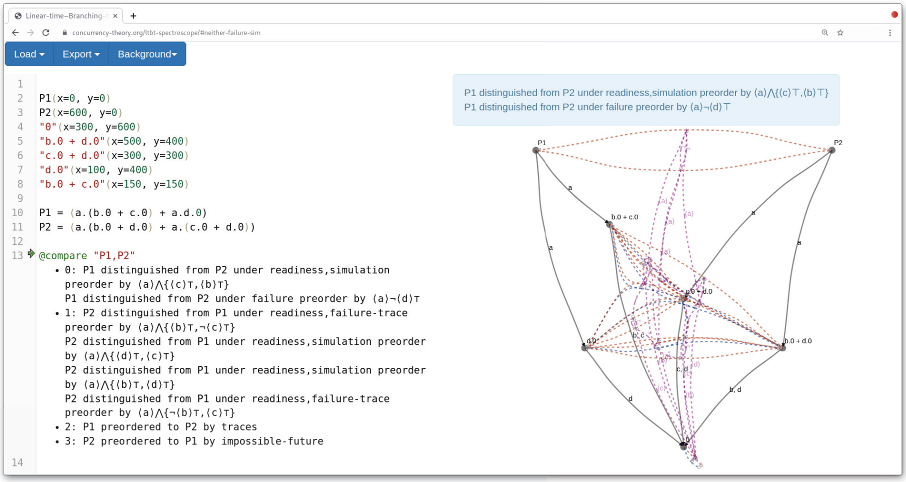

**Figure 2.** Screenshot of a linear-time–branching-time spectroscopy of the processes from Ex. 1.

to be updated. In every round, we take a game position $\mathsf{g}$ from $\mathsf{todo}$. If some of its successors have not been visited yet, we add them to the top of the work list. Otherwise we call $\mathsf{Strat}'_{F_a,\mathsf{strats}}(\mathsf{g})$ to compute distinguishing formulas using the follow-up formulas *found so far* $\mathsf{strats}$. This function mostly corresponds to Def. 14 with the twist, that partial follow-ups are used instead of recursion, and that the construction for conjunctions is split onto attacker *and* defender positions. Of the found formulas, we keep only the non-dominated ones and impossible future traces. If the result changes $\mathsf{strats}(\mathsf{g})$, we enqueue each game predecessor to propagate the update there.

The algorithm structure is mostly usual fixed point machinery. It terminates because, for each state in a finite transition system, there must be a bound on the distinguishing mechanisms necessary with respect to our metrics, and $\mathsf{Strat}'$ will only generate finitely many formulas under this bound. Keeping the impossible future formulas unbounded is alright, because they have to be constructed from trace formulas, which are subject to the bound.

## 4 A Webtool for Equivalence Spectroscopy

We have implemented the game and the generation of minimal distinguishing formulas in the "Linear-time–Branching-time Spectroscope", a Scala.js program that can be run in the browser on https://concurrency-theory.org/ltbt-spectroscope/.

The tool (screenshot in Fig. 2) consists of a text editor to input basic CCS-style processes and a view of the transition system graph. When queried to compare two processes, the tool yields the cheapest distinguishing HML-formulas it can find for both directions. Moreover, it displays the attacker-winning part of the spectroscopy game overlayed over the transition system. The latter can also enlighten matters, at least for small and comparably deterministic transition systems. From the found formulas, the tool can also infer the finest fitting preorders for pairs of processes (Fig. 3).

To "benchmark" the quality of the distinguishing formulas, we have run the algorithm on all the finitary counterexample processes from the report version of "The Linear-time–Branching-time Spectrum" [12]. Table 2 reports the output of our tool, on how to distinguish certain processes. The results match the (in)equivalences given in [12]. In some cases, the tool finds slightly better ways of distinction using impossible futures equivalence, which was not known at the time of the original paper. All the computed formulas are quite elegant / minimal.

For each of the examples (from papers) we have considered, the browser's capacities sufficed to run the algorithm in 30 to 250 milliseconds. This does not mean that one should expect the algorithm to work for systems with thousands of states. There, the exponentialities of game and formula construction would hit. However, such big instances would usually stem from preexisting models where one would very much hope for the designers to already know under which semantics to interpret their model. The practical applications of our browser tool are more on the research side: When devising compiler optimizations, encodings, or distributed algorithms, it can be very handy to fully grasp the equivalence structure of isolated instances. The Linear-time–Branching-time Spectroscope supports this process.

**Table 2.** Formulas found by our implementation for some interesting processes from [12].

| $p$ | $q$ | Cheapest distinguishing formulas found | From |
|---|---|---|---|
| P1 | P2 | $\langle a\rangle\bigwedge\{\langle c\rangle,\langle b\rangle\}\in\mathcal{O}_R\cap\mathcal{O}_S$,<br>$\langle a\rangle\neg\langle d\rangle\in\mathcal{O}_F$ | Ex. 1 |
| $a.b+a$ | $a.b$ | $\langle a\rangle\neg\langle b\rangle\in\mathcal{O}_F$ | p. 13 |
| $a.b+a.(b+c)$ | $a.(b+c)$ | $\langle a\rangle\neg\langle c\rangle\in\mathcal{O}_F$ | p. 16 |
| $a.(b+c.d)+$<br>$a.(f+c.e)$ | $a.(b+c.e)+$<br>$a.(f+c.d)$ | $\langle a\rangle\bigwedge\{\langle c\rangle\langle d\rangle,\langle b\rangle\}\in\mathcal{O}_{RT}\cap\mathcal{O}_{PF}\cap\mathcal{O}_S$,<br>$\langle a\rangle\bigwedge\{\langle c\rangle\langle d\rangle,\neg\langle f\rangle\}\in\mathcal{O}_{FT}\cap\mathcal{O}_{PF}$,<br>$\langle a\rangle\bigwedge\{\neg\langle b\rangle,\neg\langle c\rangle\langle d\rangle\}\in\mathcal{O}_{IF}$ (+3 variants) | p. 21 |
| $a.b+a.(b+c)+a.c$ | $a.b+a.c$ | $\langle a\rangle\bigwedge\{\langle c\rangle,\langle b\rangle\}\in\mathcal{O}_R\cap\mathcal{O}_S$ | p. 24 |
| $a.(b+a.(b+c.d)+$<br>$a.c.e)+a.(a.c.d+$<br>$a.(c.e+b))$ | $a.(a.(b+c.d)+$<br>$a.c.e)+a.(a.c.d+$<br>$a.(c.e+b)+b)$ | $\langle a\rangle\bigwedge\{\langle b\rangle,\langle a\rangle\bigwedge\{\langle c\rangle\langle d\rangle,\langle b\rangle\}\}\in\mathcal{O}_{RT}\cap\mathcal{O}_S$,<br>$\langle a\rangle\bigwedge\{\neg\langle b\rangle,\langle a\rangle\bigwedge\{\langle c\rangle\langle d\rangle,\neg\langle b\rangle\}\}\in\mathcal{O}_{FT}$ | p. 27 |
| $a.(b.c+b.d)$ | $a.b.c+a.b.d$ | $\langle a\rangle\bigwedge\{\langle b\rangle\langle c\rangle,\langle b\rangle\langle d\rangle\}\in\mathcal{O}_{PF}\cap\mathcal{O}_S$ | p. 31 |
| $a.b.c+a.(b.c+b.d)$ | $a.(b.c+b.d)$ | $\langle a\rangle\neg\langle b\rangle\langle d\rangle\in\mathcal{O}_{IF}$ | p. 34 |
| $a.b+a+a.c$ | $a.b+a.(b+c)+a.c$ | $\langle a\rangle\bigwedge\{\neg\langle b\rangle,\neg\langle c\rangle\}\in\mathcal{O}_F$ | p. 38 |
| $a.b.c+a.(b.c+b)$ | $a.(b.c+b)$ | $\langle a\rangle\neg\langle b\rangle\neg\langle c\rangle\in\mathcal{O}_B$ | p. 42 |

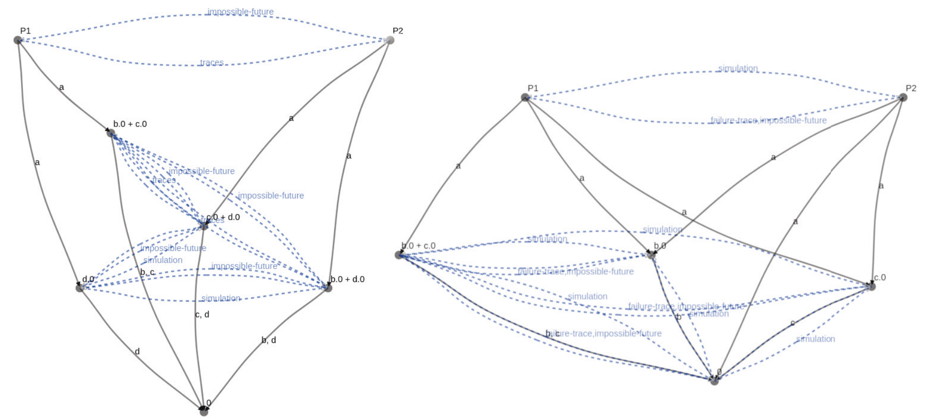

**Figure 3.** Tool output of finest preorders for transition systems. (Left: Ex. 1; right: $a.b + a.(b + c) + a.c$ vs. $a.b + a + a.c$.

## 5 Related Work and Alternatives

The game and the algorithm presented fill a blank spot in between the following previous directions of work:

**Distinguishing formulas in general.** Cleaveland [5] showed how to restore (non-minimal) distinguishing formulas for bisimulation equivalence from the execution of a bisimilarity checker based on the splitting of blocks. There, it has been named as possible future work to extend the construction to other notions of the spectrum. We are not aware of any place where this has previously been done completely. But there are related islands like the encoding between CTL and failure traces by Bruda and Zhang [7]. There is also more recent work like Jasper et. al [15] extending to the generation of characteristic invariant formulas for bisimulation classes. Previous algorithms for bisimulation in-equivalence tend to generate formulas that alternate $\langle a \rangle$ and $[b]$ observations while pushing negation to the innermost level. Such formulas can not as easily be linked to the spectrum as ours.

**Game-characterizations of the spectrum.** After Shukla et al. [18] had shown how to characterize many notions of equivalence by HORNSAT games, Chen and Deng [4] presented a hierarchy of games characterizing all the equivalences of the linear-time–branching-time spectrum. The games from [4] cannot be applied as easily as ours in algorithms because they allow word moves and thus are infinite already for finite transition systems with cycles. Constructing distinguishing formulas from attacker strategies of these games would be less convenient than in our solution. Their parametric approach is comparable to fixing maximal price budgets *ex ante*. Our on-the-fly picking of minimal prices is more flexible.

**Using game-characterizations for distinguishing formulas.** There is recent work by Mika-Michalski et al. [16] on constructing distinguishing formulas using games in a more abstract coalgebraic setting focussed on the absence of bisimulation. The game and formula generation there, however, cannot easily be adapted for our purpose of performing a *spectroscopy* also for weaker notions.

**Alternatives.** One can also find the finest notion of equivalence between two states by *gradually minimizing* the transition system with ever coarser equivalences from bisimulation to trace equivalence until the states are conflated (possibly also trying branches). Within a big tool suite of highly optimized algorithms this should be quite efficient. We preferred the game approach, because it can uniformly be extended to the whole spectrum and also has the big upside of explaining the in-equivalences by distinguishing formulas.

An avenue of optimization for our approach, we have already tried, is to run the formula search on a *directed acyclic subgraph* of the winning strategy graph. For our purpose of finding most fitting equivalences, DAG-ification may preclude the algorithm from finding the right formulas. On the other hand, if one is mainly interested in a short distinguishing formula for instance, one can speed up the process with DAG-ification by the order of remaining game rounds.

## 6 Conclusion

In this paper, we have established a convenient way of finding distinguishing formulas that use a minimal amount of expressiveness.

System analysis tools can employ the algorithm to tell their users in more detail *how equivalent* two process models are. While the generic approach is costly, instantiations to more specific, symbolic, compositional, on-the-fly or depth-bounded settings may enable wider applications. There are also some algorithmic tricks (like building the concrete formulas only after having found the price bounds and heuristics in handling the game graph) we have not explored in this paper.

So far, we have only looked at *strong* notions of equivalence [10]. We plan to verify the game in Isabelle/HOL and to extend our algorithm, so it also deals with *weak* notions of equivalence [11]. These equivalences abstract over $\tau$-actions representing “internal activity” and correspond to observation languages with a special temporal $\langle \epsilon \rangle$-observation (cf. [9]). This would generalize work on weak game characterizations such as de Frutos-Escrig et al.’s [8] and our own [2,3]. The vision is to arrive at *one* certifying algorithm that can yield finest equivalences and cheapest distinguishing formulas as witnesses for the whole discrete spectrum.

On a different note, our group is also working on an educational computer game about process equivalences.[5] The (theoretical) game of this paper can likely

[5] A prototype featuring equivalences between strong bisimulation and coupled simulation (result of Dominik Peacock’s bachelor thesis) can be played on https://www.concurrency-theory.org/rvg-game/.

be adapted to go in the other direction: from formulas to distinguished transition systems. It may thereby synthesize levels for the (computer) game. So, in the end, all this might actually contribute to actual people having actual fun.

**Acknowledgments.** We are thankful to members of our research group (especially Kim Völlinger), participants of our course Modelle Dynamischer Systeme, and the anonymous reviewers for lots of helpful comments.

## Data Availability

The source code git repository of our implementation can be accessed via https://concurrency-theory.org/ltbt-spectroscope/code/. Code to reproduce the results presented in this paper is available on Zenodo [1].
